# Hallmarks of primate lentiviral immunodeficiency infection recapitulate loss of innate lymphoid cells

**DOI:** 10.1038/s41467-018-05528-3

**Published:** 2018-09-27

**Authors:** Joseph C. Mudd, Kathleen Busman-Sahay, Sarah R. DiNapoli, Stephen Lai, Virginia Sheik, Andrea Lisco, Claire Deleage, Brian Richardson, David J. Palesch, Mirko Paiardini, Mark Cameron, Irini Sereti, R. Keith Reeves, Jacob D. Estes, Jason M. Brenchley

**Affiliations:** 1 Barrier Immunity Section, Lab of Viral Diseases, National Institute of Allergy and Infectious Diseases, National Institutes of Health, 4 Center Drive, Bethesda, MD 20892 USA; 2 AIDS and Cancer Virus Program, Frederick National Laboratory for Cancer Research, Leidos Biomedical Research, Inc, 8560 Progress Drive, Frederick, MD 21701 USA; 3 Center for Drug Evaluation and Research, Food and Drug Administration, 10001 New Hampshire Avenue, Silver Spring, MD 20903 USA; 4 Clinical and Molecular Retrovirology Section/Laboratory of Immunoregulation, National Institute of Allergy and Infectious Diseases, National Institutes of Health, 10 Center Drive, Bethesda, MD 20892 USA; 5 Department of Epidemiology and Biostatistics, Case Western Reserve University, 10900 Euclid Avenue, Cleveland, OH 44106 USA; 6 Division of Microbiology and Immunology, Yerkes National Primate Research Center, Emory University, 201 Dowman Drive, Atlanta, GA 30322 USA; 7 Center for Virology and Vaccine Research, Beth Israel Deaconess Medical Center, Harvard Medical School, 330 Brookline Avenue, Boston, MA 02215 USA; 8 Present Address: Vaccine and Gene Therapy Institute and Oregon National Primate Research Center (ONPRC), Oregon Health and Science University (OHSU), 505N.W. 185th Avenue, Beaverton, OR 97006 USA

## Abstract

Innate lymphoid cells (ILCs) play critical roles in mucosal barrier defense and tissue homeostasis. While ILCs are depleted in HIV-1 infection, this phenomenon is not a generalized feature of all viral infections. Here we show in untreated SIV-infected rhesus macaques (RMs) that ILC3s are lost rapidly in mesenteric lymph nodes (MLNs), yet preserved in SIV^+^ RMs with pharmacologic or natural control of viremia. In healthy uninfected RMs, experimental depletion of CD4^+^ T cells in combination with dextran sodium sulfate (DSS) is sufficient to reduce ILC frequencies in the MLN. In this setting and in chronic SIV^+^ RMs, IL-7Rα chain expression diminishes on ILC3s in contrast to the IL-18Rα chain expression which remains stable. In HIV-uninfected patients with durable CD4^+^ T cell deficiency (deemed idiopathic CD4^+^ lymphopenia), similar ILC deficiencies in blood were observed, collectively identifying determinants of ILC homeostasis in primates and potential mechanisms underlying their depletion in HIV/SIV infection.

## Abstract (web summary)

Innate lymphoid cells (ILCs) have been shown to be depleted during HIV-1 infection. Here the authors show that ILC loss is associated with CD4 depletion and gastrointestinal damage in a primate model of SIV infection.

## Introduction

It is widely recognized that the translocation of microbial products from a damaged gut sustained early in human immunodeficiency virus (HIV-1) infection is an important aspect of disease pathology^1–3^. Chronic gastrointestinal (GI) damage is not apparent in African nonhuman primate species that are natural hosts of simian immunodeficiency virus (SIV)^4^. Importantly, experimental GI damage in a chronic SIV-infected natural host model resulted in colitis, microbial translocation, inflammation, and CD4^+^ T cell depletion, all key pathologies resembling SIV-infected Asian macaques^5^. Indeed, GI damage in SIV-infected Asian macaques and HIV-1-infected humans results in microbial translocation that chronically stimulates the immune system and exacerbates disease progression^6^. Moreover, incomplete immune reconstitution of GI tissues in antiretroviral therapy (ART)-treated HIV-1^+^ subjects is associated with residual inflammation and heightened incidence of non-AIDS morbidities^7^. Thus, understanding the determinants of GI damage in this setting is an important step in mitigating some of the barriers that prevent HIV-1-infected subjects from returning fully to health.

Loss of interleukin-17 (IL-17)-producing and IL-22-producing CD4^+^ T cells (deemed Th17/Th22 cells) that help maintain GI integrity and anti-bacterial immunity are a determinant of GI damage, microbial translocation, and systemic immune activation in HIV/SIV infection^4,8–12^. Other IL-17/IL-22-producing cell types occupy the same anatomical niche of the GI tract, although their dynamics in HIV/SIV infection are less well studied. Innate lymphoid cells (ILCs) are one of these immune subsets. Present in GI tissues as well as other sites of the body, ILCs play critical roles in pathogen defense and tissue homeostasis^13^. While lacking antigen specificity, ILCs share many phenotypic and functional properties of adaptive immune cells. In addition to conventional natural killer (NK) cells, ILCs can be subdivided into three distinct lineages: group 1 ILCs (ILC1), ILC2s, and ILC3s, which parallel many transcriptional and functional characteristics of T helper 1 (Th1), Th2, and Th17 cells, respectively^13^. In humans, the ILC3 subpopulation can be further subdivided on the basis of NKp44 expression^14^. While ILCs are significantly outnumbered at most anatomical locations by adaptive immune cells that exert largely redundant effector functions, IL-17/IL-22-producing ILC3s and Th17/Th22 cells are relatively proportionate in the colonic mucosa of healthy uninfected humans^15^. Moreover, targeted ILC3 depletion in the presence or absence of adaptive immunity leads to dysregulated commensal bacterial containment and intestinal inflammation in mice^16,17^.

Given the importance of ILCs in GI homeostasis, several groups have studied their frequencies in progressive HIV-1 and SIV infections. In HIV-1-infected humans, ILCs in blood become apoptotic and are depleted with similar kinetics as CD4^+^ T cells^18^. ILC3 depletion of the NKp44^+^ population is also apparent in the GI tract of SIV-infected rhesus macaques (RMs)^19–21^. The mechanisms whereby ILCs are lost in HIV-1 infection are not understood, although their depletion is not likely to be a result of direct viral infection^20^. In vitro sensitivity of ILC3s to microbial Toll-like receptor (TLR)-mediated apoptosis has been proposed as a mechanism for depletion; however, no direct or correlative evidence of this finding was provided in vivo^22^, and there are conflicting evidence on whether ILCs are depleted in other human diseases characterized by dysregulated commensal microbial containment^23^. Here, we aimed to characterize ILC dynamics in nonhuman primate models of HIV infection as well as nonhuman primate models and human subjects where CD4^+^ T cells were depleted without HIV/SIV infections. We find that ILC2 and ILC3 subtypes were lost throughout SIV disease course, yet were reconstituted or preserved with pharmacologic or natural control of viremia, respectively. In both uninfected RMs experimentally depleted of CD4 T cells and human subjects with idiopathic CD4 lymphopenia (ICL), absence of CD4^+^ T cells alone was associated with severe ILC deficiencies, providing possible mechanisms of ILC loss in lentiviral immunodeficiency infections and identifying novel determinants of ILC homeostasis in health.

## Results

### ILC subpopulations can be defined in LNs of rhesus macaques

**Fig. 1:**
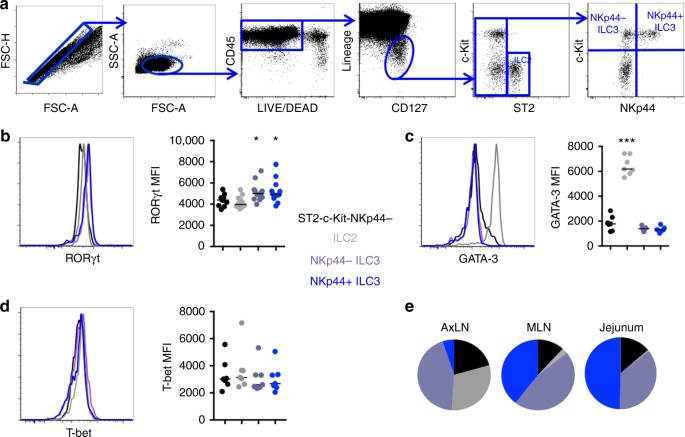
Defining ILCs in nonhuman primates. **a** Representative gating strategy for ILCs in the MLN of a healthy animal. **b** RORγT expression in MLN ILC subpopulations (*N* = 9). **c** GATA-3 expression in MLN ILC subpopulations (*N* = 7). **d** T-bet expression in MLN ILC subpopulations (*N* = 7). **e** Relative distribution of ILC subtype frequencies as a proportion of total lineage CD127^+^ cells in axillary, mesenteric lymphoid tissues, and jejunum (*N* = 7). Statistical significance was calculated using the Mann–Whitney test. ns = *P* > 0.05, * = *P* ≤ 0.05, ** = *P* ≤ 0.01, *** = *P* ≤ 0.001, and **** = *P* ≤ 0.0001

Given the importance of ILCs in GI tract barrier defense in mice, we first sought to examine whether ILC populations could be found in gut-draining MLNs of RMs. We found that lineage^−^IL7Rα^+^ ILCs constitute a small proportion of hematopoietic cells in the MLN and form distinct subpopulations that parallel those of humans and mice. c-Kit^+^NKp44^−^ and c-Kit^+^NKp44^+^ ILC3s (Fig. 1a) could be found in MLNs and expressed elevated levels of the Th17/ILC3 lineage-promoting transcription factor RAR-related orphan receptor gamma (ROR-γt) (Fig. 1b)^14,24^. Although commercially available antibodies to the human ILC2-specific marker CRTH2 did not cross-react to RMs, ILC2s could be alternatively identified by expression of the IL-33 receptor ST2 and selectively expressed the Th2/ILC2-promoting transcription factor GATA-3 (Fig. 1a, c)^25^. Putative lineage CD127^+^ ILC1 cells that lacked ST2, c-Kit, and NKp44 surface expression were present in the MLN of RMs; however, this population did not preferentially express T-bet (Fig. 1d), an important transcription factor promoting the ILC1 lineage in mice. These observations are concordant with the findings of several recent human studies^26–28^. For the purposes of this study, we have thus restricted our analysis solely to defined ILC2 and ILC3 populations. We next assessed defined ILC subtype distribution in jejunal tissue and axillary lymphoid tissue. In the jejunum, ILC2s were nearly absent, whereas NKp44^+^ ILC3s were proportionally enriched (Fig. 1e). In contrast, axillary LNs were enriched for ILC2s with very few NKp44^+^ ILC3s (Fig. 1e), highlighting a site-specific compartmentalization of ILC subtypes in nonhuman primates.

### Altered frequency of ILC subpopulations in the SIV^+^ MLN

**Fig. 2:**
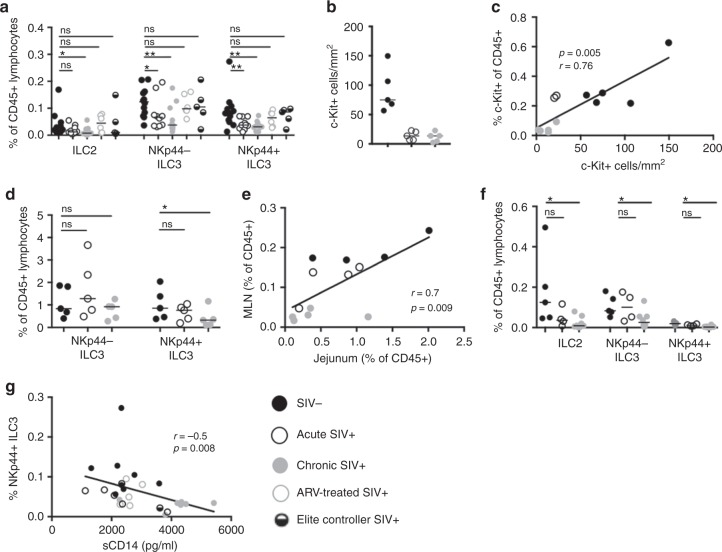
Local and systemic depletion of ILCs in untreated SIV infection. **a** Frequencies of MLN ILCs in healthy uninfected RMs (*N* = 10), untreated acute and chronic SIV-infected RMs (*N* = 10) (*N* = 11), chronic SIV^+^ RMs receiving ART (*N* = 6), and SIV-infected ECs (*N* = 4). Determined as a proportion of viable CD45^+^ hematopoietic cells. **b** Summary data of CD3-c-Kit^+^ cell number per area of paracortical region in the MLN. **c** Relationship between c-Kit^+^ LC3 proportions assessed by flow cytometry and c-Kit^+^ ILC3 enumeration by microscopy. **d** Frequencies of ILC3s in jejunal cell suspensions. **e** Correlation of NKp44^+^ ILC3 frequencies in animals with matching MLN and jejunal samples at the time of necropsy. **f** Frequencies of ILCs in axillary lymph nodes. **g** Relationship between NKp44^+^ ILC3 frequencies in the MLN and sCD14 in plasma. Statistical significance was calculated using the Mann–Whitney test. A Pearson's correlation was calculated for panels **c**, **e**, and **g**. ns = *P *> 0.05, * = *P* ≤ 0.05, ** = *P* ≤ 0.01, *** = *P* ≤ 0.001, and **** = *P* ≤ 0.0001

Depletion of gut mucosal NKp44^+^ ILC3 proportions have been reported in SIV-infected RMs^19–21^. Whether this extends to other ILC subsets in the gut is not known. To address this question, we proportionally and numerically assessed ILCs in acutely and chronically SIV-infected RMs with uncontrolled viremia, ARV-treated chronically SIV-infected RMs, and SIV^+^ elite controller (EC) RMs with natural virologic control. ILCs were defined by the gating strategy depicted in Fig. 1a. Consistent with previous findings, we confirm that depletion of MLN NKp44^+^ ILC3s occurs as early as 14 days post infection (p.i.) and is sustained in chronic infection (Fig. 2a). NKp44^−^ ILC3s were also lost in the untreated SIV^+^ MLN at similar kinetics (Fig. 2a), whereas diminished frequencies of ILC2s were observed only in the chronically, ARV-untreated, SIV-infected MLN (Fig. 2a). Frequencies of ILC3s that were lower in the untreated SIV^+^ MLN appeared to reconstitute after 6 months of ART in chronic SIV-infected animals, or were preserved in EC animals with natural control of viremia (Fig. 2a). We additionally assessed SIV-associated alterations in cells sharing a similar ILC3-defining surface phenotype per area of tissue by quantitative fluorescence microscopy. We specifically enumerated c-Kit^+^ nucleated cells with a lymphocytic morphology that lacked expression of CD3, which were found to largely localize to the T cell-rich paracortical region of the LN (Supplementary Figure 1). In the untreated acute and chronic SIV^+^ MLN, CD3^−^c-Kit^+^ cell numbers were significantly diminished (6.5–8-fold reduction) when compared to numbers of these cells in MLNs of healthy uninfected animals (Fig. 2b). Importantly, CD3^−^c-Kit^+^ cell numbers correlated directly with proportional assessment of c-Kit^+^ ILC3s in the MLN by flow cytometry (Fig. 2c).

To assess how alterations of ILC frequencies in gut-draining MLNs are reflective of the GI tract itself, we assessed their relative proportions in the jejunum of uninfected and untreated SIV^+^ RMs. While not apparent in acutely SIV-infected RMs, NKp44^+^ (but not NKp44^−^) ILC3 frequencies were diminished in the jejunum of chronic SIV^+^ RMs (Fig. 2d). Jejunal NKp44^+^ ILC3 frequencies of uninfected and SIV-infected RMs paralleled that of the MLN (Fig. 2e), suggesting SIV-associated depletion of these cells are unlikely due to altered migration or retention in intestinal tissues. ILC2 and ILC3s in chronic SIV^+^ RMs were also diminished in AxLNs that are distal to the GI tract (Fig. 2f). Importantly, frequencies of MLN NKp44^+^ ILC3s in our cohort correlated with soluble CD14 (sCD14) levels in plasma, a predictor of non-AIDS morbidities in treated HIV-1^+^ subjects (Fig. 2g)^7^.

### Virologic control rescues SIV-associated defects in ILCs

**Fig. 3:**
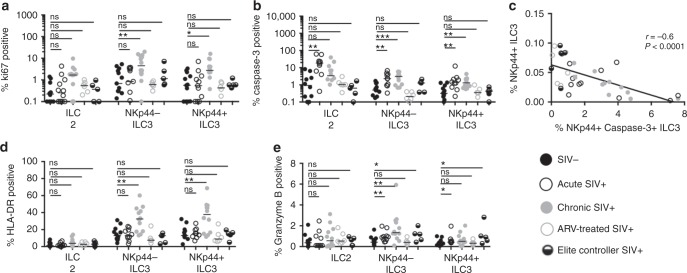
ILC dysfunction in the SIV^+^ MLN normalizes with ART or elite control. **a** Frequencies of MLN ILCs in cell cycle in healthy uninfected RMs, untreated acute, and chronic SIV-infected RMs, chronic SIV^+^ RMs receiving ART, and SIV-infected ECs. **b** Frequencies of MLN ILCs shown to express active caspase-3 in the uninfected and SIV^+^ MLN. **c** Relationship between NKp44^+^ ILC3 frequencies in the MLN and NKp44^+^ ILC3s expressing active caspase-3. **d** Percentage of MLN ILCs expressing HLA-DR. **e** Percentage of MLN ILCs expressing granzyme B. Statistical significance was calculated using the Mann–Whitney test. A Pearson's correlation was calculated for panel (**c**). ns =* P* > 0.05, * =* P* ≤ 0.05, ** = *P* ≤ 0.01, *** = *P* ≤ 0.001, and **** = *P* ≤ 0.0001

We next examined rates of cellular cycling and death by measuring intracellular expression of ki67 and active caspase-3. In MLNs of healthy uninfected animals, ILCs were relatively quiescent with very few cells seen to be in cycle or undergoing apoptosis (Fig. 3a, b), yet in the chronic SIV^+^ MLN, NKp44^−^ and NKp44^+^ ILC3s expressing ki67 were found to be elevated (Fig. 3a). Frequencies of MLN NKp44^−^ and NKp44^+^ ILC3s in cell cycle appeared to normalize in SIV^+^ RMs with pharmacological control of viremia and were not different in EC RMs with natural control of viremia (Fig. 3a). ILC3s in MLNs of untreated SIV^+^ RMs (but not in MLNs of ART-treated or EC RMs) expressed higher levels of active caspase-3 (Fig. 3b). Moreover, the frequencies of NKp44^+^ ILC3s in MLNs of all SIV^+^ RMs correlated inversely with active caspase-3 expression in this subset, suggesting that loss of this subset may be due to apoptotic death (Fig. 3c),

We next assessed the activation state of ILCs in the SIV^+^ MLN by surface expression levels of HLA-DR. HLA-DR expression was mainly restricted to NKp44^−^ and NKp44^+^ ILC3 subtypes in the healthy uninfected MLN (Fig. 3d). In the untreated SIV^+^ MLN, HLA-DR surface expression was elevated on both NKp44^−^ and NKp44^+^ ILC3s s in chronically but not acutely SIV-infected RMs, and were either normalized or preserved in MLN ILC3s of ART-treated or EC animals, respectively (Fig. 3d). We also assessed intracellular granzyme B expression, a surrogate marker of cytotoxicity. Recent observations have indicated that NKp44^+^ ILC3s can acquire cytotoxic potential in response to chronic inflammatory conditions^21^. In line with these findings, granzyme B expression was elevated in both NKp44^+^ and NKp44^−^ ILC3s in the acute SIV^+^ MLN (Fig. 3e). While not different in MLN ILCs of chronic SIV^+^ RMs receiving ART, ECs tended to exhibit elevated intracellular expression of granzyme B in NKp44^−^ and NKp44^+^ ILC3s (Fig. 3e). In contrast to the ILC3 subtype, granzyme B expression was not observed in ILC2s in MLNs of uninfected or SIV^+^ animals (Fig. 3e).

### ILC2s are functionally impaired in the SIV^+^ MLN

**Fig. 4:**
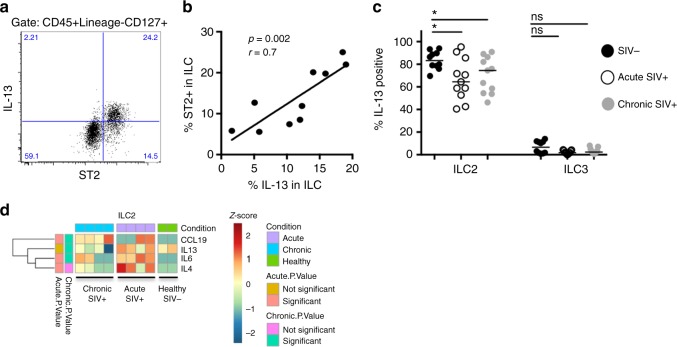
IL-13 production is impaired in ILC2 cells in MLNs that are marked by ST2 expression. **a** Plot of ST2 expression in total ILC compartment against IL-13 production in total ILC compartment. **b** Correlation between ST2^+^ ILCs and IL-13-producing ILCs in a cohort of uninfected animals. **c** Mes LNMCs from healthy, acute, or chronically SIV-infected animals were stimulated for 6 h with PMA/ionomycin. Intracellular IL-13 expression was measured in ILC1, ILC2, and ILC3 subtypes. **d** Gene expression profiles of cytokines produced by ILC2. Color scheme in both heatmaps represents the number of standard deviations above (red) or below (blue) the mean. Statistical significance was determined by the Mann–Whitney test. A Spearmann's correlation was calculated for panel **b**. ns = *P* > 0.05, * = *P* ≤ 0.05, ** = *P* ≤ 0.01, *** = *P* ≤ 0.001, and **** = *P* ≤ 0.0001

Several reports in humans have observed that deregulation of the ILC2 subtype is associated with a number of Th2-driven airway diseases^29–31^. Little is known how ILC2 function is affected during chronic viral infections, which characteristically induce Th1-biased immune responses. To explore this question in the context of SIV infection, we stimulated MLN cell suspensions from RMs with phorbol 12-myristate 13-acetate (PMA) and ionomycin and assessed intracellular IL-13 production. We found that cells induced to make IL-13 in the MLN were selective to those that expressed the IL-33 receptor ST2 (Fig. 4a). Moreover, IL-13 production in total ILCs directly correlated with frequencies of ST2^+^ ILCs in healthy animals (Fig. 4b), indicating that ST2 expression can reliably define IL-13-producing ILC2s in tissues of nonhuman primates. In the untreated SIV^+^ MLN, production of this cytokine was diminished in ILC2s at both the acute and chronic stage (Fig. 4c). This was also apparent in transcriptional profiling of ILC2s. Transcripts encoding for IL-13 in unstimulated ILC2s of healthy uninfected RMs displayed spontaneous production of this cytokine, and IL-13 transcript levels were reduced in ILC2s from the chronic, but not acute SIV^+^ MLN (Fig. 4d).

### Heightened IL-17A production In ILC3s of the SIV^+^ MLN

**Fig. 5:**
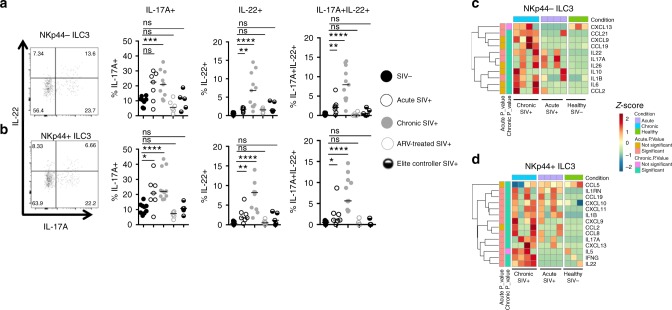
SIV infection is associated with significant functional changes in MLN ILCs. Mesenteric lymph node mononuclear cells from healthy (*N* = 7), acute (*N* = 7), chronic (*N* = 9), ART-treated (*N* = 6), or EC SIV-infected animals (*N* = 5) were stimulated for 6 h with PMA/ionomycin. Intracellular IL-17A and IL-22 expression was measured in NKp44^−^ (**a**) or NKp44^+^ (**b**) ILC3. Gene expression profiles of selected cytokines and chemokines produced by NKp44^−^ (**c**) and NKp44^+^ (**d**) ILC3s in the SIV^−^ (*N* = 3) and the untreated acute (*N* = 4) and chronic (*N* = 4) SIV^+^ MLN. Color scheme in both heatmaps represents number of standard deviations above (red) or below (blue) the mean. Statistical significance was determined by the Mann–Whitney test for **a**, **b**. Statistical significance of **c**, **d** was determined using the Wald test with Bonferroni correction for multiple comparisons. ns = *P* > 0.05, * = *P* ≤ 0.05, ** = *P* ≤ 0.01, *** = *P* ≤ 0.001, and **** = *P* ≤ 0.0001

Given the importance of IL-17 and IL-22 in GI homeostasis, we next examined the function of MLN-resident ILC3s that are enriched for production of these cytokines. Consistent with previous reports^11,32^, Th17 and Th22 cell function was characteristically diminished in the untreated SIV^+^ MLN, and remained diminished in MLNs of chronic SIV^+^ animals receiving ARVs and EC animals, with the exception of preserved Th22 cell frequencies in EC animals (Supplementary Figure 2a, b). While overall frequencies of ILC3s were decreased, the ability of remaining ILC3s to produce IL-17, IL-22, or both cytokines when stimulated were significantly elevated in the untreated acute and chronic SIV^+^ MLN (Fig. 5a, b). The only exception was IL-17 single-producing NKp44^−^ ILC3s in the acute SIV^+^ MLN (Fig. 5a, b). In contrast, no differences were observed in single-producing or double-producing IL-17/IL-22 ILC3s of ARV-treated or EC animals in the MLN (Fig. 5a, b), indicating that aberrant IL-17 and IL-22 production by ILC3s is a feature of untreated SIV infection, yet normalizes with pharmacological or natural control of viremia.

Transcriptomic profiling revealed low abundance of IL-17 and IL-22 transcripts and a relatively quiescent state of NKp44^−^ and NKp44^+^ ILC3s in the healthy uninfected MLN (Fig. 5c, d). In the untreated SIV^+^ MLN, gene expression of numerous cytokine transcripts was significantly up-regulated, particularly in chronically infected RMs (Fig. 5c, d). IL-17/IL-22 gene expression was increased in NKp44^−^ and NKp44^+^ ILC3 of the SIV^+^ MLN (Fig. 5c, d), in line with our functional observations. There were also clear distinctions in cytokine transcript profiles between NKp44^−^ and NKp44^+^ subtypes in the chronic SIV^+^ MLN. Transcripts encoding IL-10 and IL-26 were selectively expressed in NKp44^−^ ILC3s, whereas NKp44^+^ ILC3s selectively expressed interferon (IFNγ) and IL-5 transcripts (Fig. 5c, d). Interestingly, up-regulation of IFNγ transcripts was associated with a stable c-Kit^+^NKp44^+^ surface phenotype in MLN ILC3s, suggesting that IFNγ secretion by ILC3 in vivo may not require loss of these surface markers, as opposed to what has been observed of functional switches induced in ILC3s in vitro^33–35^.

### Loss of ILCs in CD4-depleted, DSS-treated uninfected RMs

**Fig. 6:**
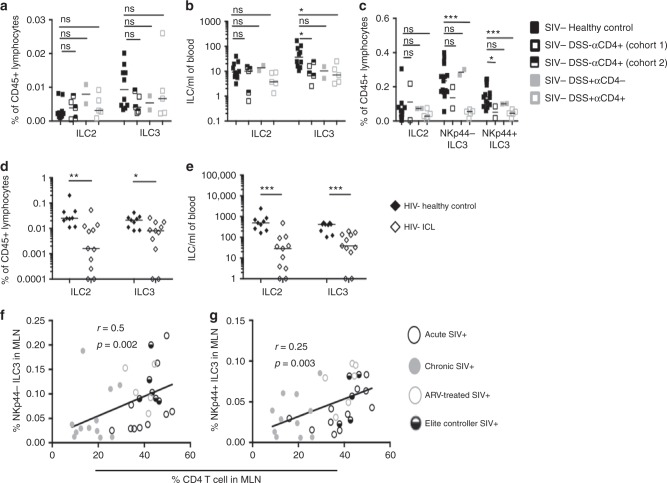
Deficient ILCs in CD4 lymphopenic HIV/SIV-uninfected human and nonhuman primates. **a** Frequencies and **b** absolute numbers of ILC subsets in blood of uninfected control animals (*N* = 9), animals receiving DSS (*N* = 2), or animals experimentally depleted of CD4 T cells with (*N* = 5) or without DSS treatment (*N* = 5). **c** Frequencies of ILCs in MLN of healthy control, SIV-uninfected DSS-treated, αCD4-treated, or animals receiving both treatments. **d** Frequencies and **e** absolute numbers of blood ILCs in healthy (*N* = 10) and ICL (*N* = 11) human subjects. Correlation between **f** NKp44^−^ ILC3 and **g** NKp44^+^ ILC3 percentages and CD4 T cell percentage in the MLN of SIV^+^ RMs. Statistical significance was calculated using the Mann–Whitney test. A Pearson's correlation was calculated for panels **f**, **g**. ns = *P* > 0.05, * = *P* ≤ 0.05, ** = *P* ≤ 0.01, *** = *P* ≤ 0.001, and **** = *P* ≤ 0.0001

The fact that ILCs are not permissive to HIV/SIV infection prompted us to explore factors other than direct viral infection that may contribute to HIV/SIV-associated ILC loss^20^. To recapitulate hallmarks of HIV/SIV pathology in a setting devoid of SIV replication, we examined two cohorts of uninfected RMs treated with a CD4-depleting antibody (αCD4). In the second cohort of CD4-depleted RMs, some of these animals were treated with DSS, which induces a low-grade endotoxemia and recapitulates aspects of pathologic SIV/HIV-1 infection^5^. In animals receiving αCD4 alone or in combination with DSS, circulating numbers of CD4 T cells were significantly reduced, yet ILCs were not reduced in animals receiving a control IgG antibody or DSS only (Supplementary Figure 3a). αCD4 treatment did not have similarly dramatic effects on other populations of lymphocytes (Supplementary Figure 3b). Proportions of blood ILCs were similar among these treatment groups (Fig. 6a). In contrast, absolute numbers of blood ILC3s (but not ILC2s) were significantly diminished in RMs receiving αCD4 alone or in combination with DSS, yet unchanged in DSS-only-treated RMs (Fig. 6b). We also examined the effect of CD4 depletion and DSS treatment on proportions of tissue-resident ILCs in the MLN. MLNs of αCD4-treated RMs, but not DSS-only-treated RMs, were significantly depleted of CD4 T cells (Supplementary Figure 4c). Although ILC frequencies among healthy control RMs and DSS-treated RMs receiving control IgG were comparable (Fig. 6c), frequencies of MLN NKp44^+^ ILC3s were decreased in αCD4-only-treated RMs, reaching statistical significance despite a limited sample size. In a larger group of CD4-depleted RMs receiving DSS, frequencies of both NKp44^−^ and NKp44^+^ ILC3s were dramatically reduced in the MLN (Fig. 6c), whereas ILC2 frequencies were not affected (Fig. 6c).

To determine whether similar mechanisms may be operable in humans, we turned to a cohort of HIV-uninfected subjects characterized by sustained circulating CD4^+^ T cell counts below 300 cells/μl, termed ICL^36^. A summary of absolute CD4, CD8, and NK cell counts in blood of control and ICL cohorts is provided in Supplementary Table 1. At the time of study, some of these subjects presented some form of infectious complication, while others were asymptomatic (Supplementary Table 1). To study GI barrier dysfunction in this cohort, we measured plasma levels of intestinal fatty-acid-binding protein (IFAPB) and sCD14^37^. In line with previous studies, ICL subjects exhibited significant elevation of sCD14, yet comparable levels of IFABP when compared to plasma levels of these proteins in healthy control subjects (Supplementary Figure 3d). We next assessed ILCs in blood of ICL subjects, defining them in a similar fashion as other human studies (Supplementary Figure 3e)^18,34^. In concordance with SIV-uninfected RMs receiving αCD4 in the presence or absence of DSS, ICL subjects exhibited decreased proportions of ILC3s in blood and additional reductions of blood ILC2s (Fig. 6d). ICL subjects had dramatically fewer absolute numbers of blood ILC2 and ILC3s when compared to ILC numbers in blood of healthy subjects (Fig. 6d, e and Supplementary Table 2). In two particular ICL subjects, ICL1 and ICL10, ILCs were completely absent from blood (Supplementary Table 2 and Fig. 6c, d). In consideration of alternative scenarios, we surmised that this phenomenon could be due to developmental defects in a common precursor shared between CD4^+^ T cells and ILCs. The most proximal precursor shared by these two lineages is the common lymphoid progenitor (CLP), and CD8^+^ T cells (which also arise from the CLP) would also be affected in this case. We thus stratified our ICL cohort based on circulating CD8^+^ T cell counts (Supplementary Table 1), yet found no differences in the number of blood ILCs between ICL subjects that were CD8 lymphopenic, had normal or abnormally expanded CD8^+^ T cells (Supplementary Figure 3f), arguing against this scenario. Given the observed relationship between CD4 and ILC deficiencies in ICL subjects, we also assessed the relationship between frequencies of these two cell types in the SIV^+^ MLN. Indeed, we observed a direct correlation in the SIV^+^ MLN with CD4 T cells and both NKp44^−^ and NKp44^+^ ILC3s (Fig. 6f, g). Thus, even in settings without HIV/SIV infections, key features of primate lentiviral immunodeficiency disease are marked by depletion of ILCs in both blood and lymphoid tissues.

### Features of HIV-1/SIV infections diminish IL-7R on ILCs

**Fig. 7:**
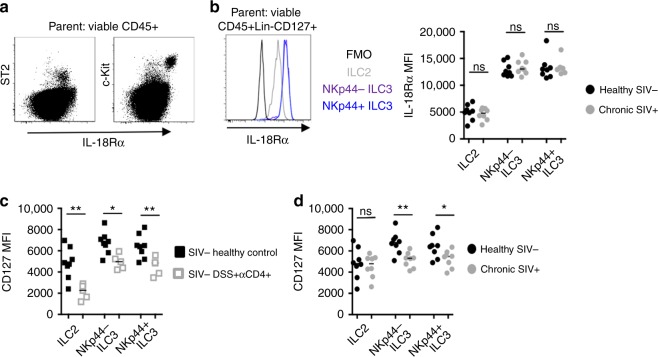
CD4 depletion in RMs reduces CD127 surface expression on MLN ILCs. **a** Representative dot plots of IL-18Rα expression on ST2 and c-Kit-expressing hematopoietic cells in the MLN. **b** MLN ILCs were defined by CD127 surface expression and IL-18Rα MFI was assessed on MLN ILCs from healthy uninfected (*N* = 8) and chronic SIV^+^ RMs (*N* = 7). **c** ILCs were defined by IL-18Rα surface expression and CD127 MFI were assessed on MLN ILCs from SIV^−^ healthy control (*N* = 8) and DSS^+^ αCD4^+^ RMs (*N* = 5). **d** Summary data of CD127 surface expression on IL-18Rα-expressing ILCs in the SIV-uninfected and chronic SIV^+^ MLN (*N* = 7). Statistical significance in panels **b**–**d** were calculated using the Mann-Whitney test. ns = *P* > 0.05, * = *P* ≤ 0.05, ** = *P* ≤ 0.01, *** = *P* ≤ 0.001, and **** = *P* ≤ 0.0001

Our observations of ILC loss in multiple settings of CD4 deficiency prompted us to explore how features of HIV-1/SIV regulate factors controlling ILCs maintenance. We focused on the expression of the γ-chain cytokine receptor IL-7Rα (CD127), known for its importance on ILC homeostasis in both mice and humans^26,38^. Thus far, it has been difficult to determine whether disease states are associated with altered CD127 surface expression, as ILCs themselves are universally defined by their high surface expression of this molecule. We thus sought to identify alternative “pan-ILC” markers that were stably expressed by all defined ILC subpopulations in the MLN. A previous study has found that both ILC2 and ILC3 subtypes in human tissues express the IL-18Rα chain and are responsive to IL-18 in vitro^27^. In MLNs of nonhuman primates, IL-18Rα was found on the surface of ST2-expressing ILC2s (Fig. 7a). c-Kit^+^ ILC3s in particular expressed IL-18Rα at levels higher than all other hematopoietic cell types in the MLN (Fig. 7a). As in CD127^+^ ILCs, the lineage-defining transcription factors GATA-3 and RORγt were selectively enriched in IL-18Rα^+^ ILC2 and ILC3 subtypes, respectively (Supplementary Figure 4a, b). Importantly, when defining ILCs by CD127, surface densities of IL-18Rα were not altered in the chronic SIV^+^ MLN (Fig. 7b). In contrast, CD127 surface densities when defining ILCs by IL-18Rα were significantly reduced in two settings of CD4 T cell deficiency. This was true of both ILC2 and ILC3 subtypes in SIV-uninfected RMs receiving αCD4 and DSS (Fig. 7c), and was also evident on ILC3s in the chronic SIV^+^ MLN (Fig. 7d). Reductions in CD127 surface expression did not result in a complete loss of this molecule, as ILC3s in the chronic SIV^+^ MLN all remained positive for CD127 expression (Supplementary Figure 5a, b), and NKp44^+^ ILC3 frequencies continued to be diminished in the SIV^+^ MLN when defined by IL-18Rα^+^ expression (Supplementary Figure 5c). Thus, while ILCs express high levels of both CD127 and IL-18Rα, CD127 appears to be less stable during SIV infection or when features of SIV infection are induced experimentally in uninfected RMs with αCD4 and DSS treatment.

### NKp44^+^ ILC3s in the MLN are highly responsive to type I IFN in vivo

**Fig. 8:**
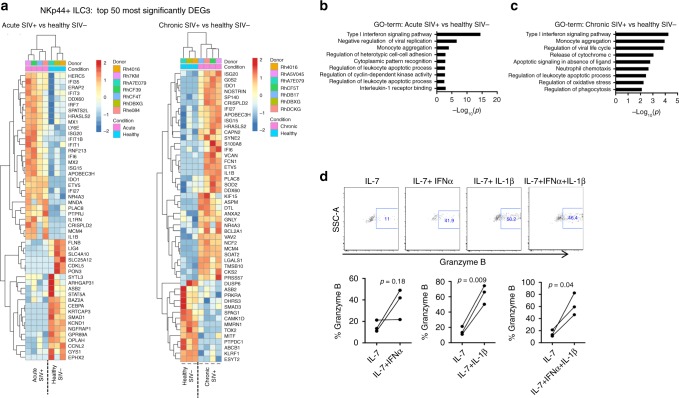
NKp44^+^ ILC3s exhibit robust IFN gene signatures in the SIV^+^ MLN. **a** Gene expression profiles of the top 50 most significantly DEGs among NKp44^+^ ILC3s in the acute (*N* = 3) and chronic SIV^+^ MLN (*N* = 4). Color scheme represent standardized gene expression (*z*-score) with red and blue signifying up-regulated and down-regulated genes, respectively. The list of top 50 DEGs in **a** were functionally annotated by GO term analysis for significantly enriched pathways in acute SIV^+^ (**b**) and chronic SIV^+^ contrasts (**c**). Significance was determined by a Fisher’s exact test on the likelihood of their association compared to other genes in the gene universe. **d** ILC subpopulations from MLNs of healthy animals (*N* = 3) were sorted and stimulated with IL-7 in the presence or absence of IL-1β and/or IFNα. Intracellular granzyme B was assessed following 6 days of culture. Significance was determined using the paired Student’s *t* test. ns = *P* > 0.05, * = *P* ≤ 0.05, ** = *P* ≤ 0.01, *** = *P *≤ 0.001, and **** = *P* ≤ 0.0001

To gain insight into gene signatures associated with SIV-associated ILC loss, we analyzed genome-wide transcriptomic profiles from NKp44^+^ ILC3s sorted by an identical gating strategy to Fig. 1a. Significant transcriptional changes were observed in NKp44^+^ ILC3s as early as day 14 p.i. and persisted in the chronic SIV^+^ MLN. Among these transcriptional alterations, we selected a representative dataset comprising the 50 most significantly differentially expressed genes (DEGs) in NKp44^+^ ILC3s (Fig. 8a). These DEGs were functionally annotated by gene ontology terms. In NKp44^+^ ILC3s of the acute SIV^+^ MLN, genes involved in type I IFN signaling were found to be the most significantly enriched, followed by genes regulating cell–cell adhesion (*NR4A3*, *IL1B*) (Fig. 8b). Interestingly, type I IFN gene signatures coincided with enrichment of cellular division gene pathways regulating cyclin-dependent protein kinase activity (*CCNL2*, *CEBPA*, *HERC5*), and genes regulating apoptosis (*IDO1*, *NR4A3*) (Fig. 8b). In the acute SIV^+^ MLN, genes associated with IL-1 receptor binding were also enriched in NKp44^+^ ILC3s (*IL1B*, IL1RN) (Fig. 8b). Type I IFN signaling was also the most significantly represented gene pathway in NKp44^+^ ILC3s of the chronic SIV^+^ MLN (Fig. 8b), coinciding with genes regulating apoptosis (*IDO1*, *NR4A3*), release of cytochrome *c* (*BCL2A1*, *IFI6*, *SOD2*), and cellular responses to oxidative stress (*ETV5*, *SOD2*) (Fig. 8b).

Given the observed gene signatures of type I IFN and IL-1 exposure in NKp44^+^ ILC3s of the SIV^+^ MLN, we asked whether in vitro treatment of ILC3s with these cytokines could recapitulate observed ILC3 phenotypes associated with SIV infection. While marginal effects of these cytokines on cell cycling and death rates were observed, both IFNα and IL-1β were potent inducers of proteins involved in cytotoxicity. A small fraction of purified NKp44^+^ ILC3s expressed granzyme B with IL-7 alone after 6 days in culture, yet the addition of IL-1β or in combination with IFNα significantly up-regulated granzyme B expression (Fig. 8c). IFNα treatment alone also increased the cytotoxic potential in two of three animals assessed (Fig. 8c). Thus, in vitro exposure to known antiviral and proinflammatory factors associated with progressive SIV/HIV-1 infection can recapitulate cytotoxic phenotypes of NKp44^+^ ILC3s observed directly ex vivo in the SIV^+^ MLN.

## Discussion

In humans, disease states marked by chronic GI inflammation are associated with ILC deregulation. Chronic GI inflammation is a hallmark of HIV-1 and progressive SIV infection^7^, and recent evidence indicates that death of blood ILCs occurs early in HIV-1 disease course. There is reason to suspect that ILCs are important in HIV-1 pathology, as we observe, also corroborated by others, that loss of ILCs are associated with elevated levels of sCD14 and other systemic inflammatory markers^15,18,39^. Here we also characterized the ILC2 subtype. To our knowledge, this represents the first characterization of these cells in nonhuman primate species. We also show that IL-18Rα is a reliable pan-ILC marker in primates. By extension, we found that IL-18Rα surface levels remain stable on ILCs in the SIV^+^ MLN, while surface expression of the widely used pan-ILC marker CD127 diminish in both the SIV^+^ MLN and in MLNs of uninfected RMs treated with αCD4 and DSS. Similar reductions of CD127 are well established in CD4 and CD8 T cells of untreated and ART-treated HIV-infected subjects^40,41^. While it is unknown if CD127 surface expression is similarly reduced on ILCs in other disease settings, these findings suggest that it may be important to consider the context when using CD127 as a pan-ILC marker, particularly during chronic inflammatory conditions.

At steady state, we found that ILCs in the SIV-uninfected MLN are relatively quiescent with low rates of cellular turnover. In the ARV-untreated SIV^+^ MLN, however, cellular cycling and apoptosis of all ILC subtypes are elevated and the ILC3 population displays heightened expression of HLA-DR, granzyme B, and elevated production of IL-17 and IL-22. We did not observe these alterations in animals with pharmacological or natural control of viremia. These findings in total point to an early loss of ILC3s and generalized state of ILC3 activation in the untreated SIV^+^ MLN that is not apparent in settings of viremic control. Whether these observations are concordant with ILC dynamics and function in tissues of HIV-1^+^ humans is currently unclear. In a small cohort of HIV-1^+^ subjects on ART, one study has observed frequencies of ILC3s to be decreased in the colon but not at other anatomical sites of the GI tract^39^. Another study that observed early and durable depletion of ILC numbers in blood of untreated HIV-1^+^ subjects found the frequencies and functionality of these cells to be preserved in tonsils and the colon^18^. In each of these cases, an unresolved question is whether proportional assessments are fully representative of true ILC numbers at mucosal sites, and quantitative immunohistochemical approaches may shed further insight into this important issue. Importantly, we observe that enumeration of CD3^−^c-Kit^+^ cells in the MLN by IHC correlates significantly with proportional assessment of c-Kit^+^ ILC3s. Although we cannot rule out that a CD3^−^c-Kit^+^ surface phenotype defines ILC3s exclusively, we can be reasonably certain in our study that reduced ILC3 frequencies in the SIV^+^ MLN observed by flow represent a true loss of these cells. It is also likely that loss of ILC3s at this site, while representing a small percent of hematopoietic cells in the MLN, are biologically significant. Indeed, we have observed that IL-17-producing ILCs both in the GI tract and gut-draining MLNs correlate directly with physical breaches to the GI barrier in SIV^+^ RMs^32^.

Drastic depletion of ILCs is not a generalized feature of the acute-phase response to viral infections^18,42^. Thus, there is considerable interest regarding the exact mechanisms of ILC loss in HIV-1/SIV infection. In two settings devoid of SIV/HIV-1 infection, we show here that CD4 T cell deficiency is associated with depletion of ILCs in the blood and MLN. This was true in healthy nonhuman primates experimentally depleted of CD4 T cells and human subjects with ICL, a presumably heterogeneous syndrome that, regardless of the upstream mechanisms, results in profound CD4 deficiency^36^. While a notable difference between these two settings of CD4 lymphopenia and HIV-1/SIV infection is the lack of an infectious component, these data may shed novel insights into ILC biology, and offer striking parallels to ILC dynamics in HIV-1/SIV infection. Interestingly, there is indeed some precedence for these findings in other species. ILC2s in the lung of antigen-experienced mice were significantly reduced upon treatment with an αCD4-depleting antibody^43^. Nevertheless, a key caveat to our study is that we cannot rule out the role of GI damage in mediating some of these observations. Only two animals in the αCD4/DSS study were included in the DSS-only-treated group, and measurements of sCD14 (but not IFABP) were elevated in plasmas of ICL subjects. In disease settings of GI dysfunction without overt CD4 depletion such as Crohn’s disease or ulcerative colitis, ILC frequencies in the gut of these particular subjects were either increased or unchanged, respectively^44^. DSS treatment of mice also induces expansion rather than depletion of ILC3s in the gut^45^. Thus, two important questions yet to be answered from our study include^1^ how GI damage or CD4 depletion are independently responsible for the observed loss of ILCs and^2^ the mechanisms that may underlie potential cross-talk between CD4 T cells and ILCs.

Transcriptomic analysis of NKp44^+^ ILC3s in the SIV^+^ MLN showed strong signatures of IFNα and/or IL-1β exposure, and we show here that treatment with these cytokines in vitro can regulate NKp44^+^ ILC3 cytotoxic potential through up-regulation of granzyme B. Whether this translates to direct antiviral activity in vivo is currently unclear. A recent report has indicated that NKp44^+^ ILC3s in rectal tissues are associated with delayed SIV acquisition in vaccinated RMs challenged with SIVmac251^46^, suggesting a plausible role for NKp44^+^ ILC3s in direct antiviral defense. Given that local IL-1β and IFNα induction in vaginal tissues precede detectable viremia in early stages of SIV disease course^47^, it will be interesting to assess whether potentially cytotoxic NKp44^+^ ILC3s can be found at this site of HIV-1/SIV transmission. To date, one study has examined NKp44^+^ ILC3s in the vaginal mucosa, yet in uninfected animals the frequencies of these cells were significantly lower than at other mucosal sites^48^. In recent murine studies, the ILC1 population has been shown to confer host protection at initial sites of viral infection^49^. These cells display Th1-like profiles, are lineage-negative and express CD127, yet lack ILC2-defining and ILC3-defining surface markers. We chose, however, not to include an analysis of this population in our study given that there are no currently available markers to accurately identify them. Indeed, RNAseq analysis of lineage^−^CD127^+^ “ILC1s” revealed that this population expressed markers associated with non-ILCs (including CD3), similar to previous reports in humans^27,28^. Importantly, we cannot conclude that a cell population analogous to the well-characterized ILC1s in mice does not exist in humans and nonhuman primates. Only that interpretations drawn from this population should be done with caution given its apparent heterogeneity in primate species.

In summary, we provide a fairly comprehensive report on tissue-resident ILC subtype dynamics in SIV infection and in animals wherein hallmarks of HIV/SIV disease pathogenesis are recapitulated. Given their functional overlap with certain adaptive immune subsets, the overarching question of whether ILCs in primates are biologically important in health or disease is still unclear. In a cohort of humans with severe combined immunodeficiency, ILCs were severely deficient, yet even over prolonged periods of time, susceptibility to any particular disease was not observed in these patients^26^. In contrast, most ICL patients in our cohort exhibited some form of clinical manifestation at the time of study (Supplementary Table 1). As both CD4^+^ T cell loss and GI damage appear to contribute to ILC depletion in SIV infection, strategies that either enhance CD4^+^ T cell reconstitution or target GI reconstitution may hold promise an improve the prognosis of individuals with inflammation due to GI tract abnormalities.

## Methods

### Nonhuman primate animals

This study was performed with 10 acutely SIV-infected (14 days p.i.) RMs (*Macaca mulatta)*, 12 chronically SIV-infected (day 90+ p.i.) RMs, and 13 SIV-uninfected RMs. All RMs in this study were of mature Indian origin consisting of male animals with an age range of 2.5–8 years. All RMs used in this study were infected intravenously with SIV_mac239_, with the exception of one SIV^+^ animal infected chronically with SIV_smE543_. SIV_mac239_ virus stock was obtained by transfection of 293T cells and titrated on TZM-bl cells. The SIV_smE543_ was derived from a terminal isolate from animal RhE543^50^. In studies in ART-treated animals, six RMs were infected intrarectally with 10,000 TCID50 of SIVmac239. Six weeks post infection ART was initiated, consisting of a regimen of 20 mg/kg per day PMPA/Tenofovir, 40 mg/kg per day FTC/Emtricitabine, 2.5 mg/kg per day Dolutegravir, and 375 mg Darunavir. EC animals were defined as having viral set points below limits of detection. EC animals were inoculated with SIVsmE660 clone that had been mutated to be resistant to TRIM 5. The SIVsmE660 was prepared from virus stock generated by growth in pig-tailed macaque peripheral blood mononuclear cells (PBMCs)^51^. Four of the five EC animals possessed the MAMU A*01 MHC allele. For the CD4^+^ T cell depletion experiments, we treated SIV-uninfected RMs with eight treatments of rhesus recombinant CDR-grafted anti-CD4 antibody (denoted as cohort 2) or control rhesus IgG1 (50 mg/kg SQ; NIH Nonhuman Primate Reagent Resource) every 3 weeks with or without six cycles of DSS treatment (1 cycle = 2 weeks on DSS followed by 2 weeks off DSS). CD4^+^ T cell depletion in a second cohort of RMs (denoted as cohort 1) was performed with four separate treatments of 10 mg/kg intravenous anti-CD4 mAb (clone OKT4A), spaced 3 days apart. Blood from this cohort was collected at day 120 post CD4 depletion. These studies were carried out in strict accordance with the recommendations described in the Guide for the Care and Use of Laboratory Animals of the National Institutes of Health, the Office of Animal Welfare, and the United States Department of Agriculture. All animal work was approved by the NIAID Division of Intramural Research Animal Care and Use Committees (IACUC) in Bethesda, Maryland (protocols LPD-26 and LMM-6), and the National Cancer Institute (Assurance #A4149-01). The animal facility is accredited by the American Association for Accreditation of Laboratory Animal Care. All procedures were carried out under ketamine anesthesia by trained personnel under the supervision of veterinary staff, and all efforts were made to maximize animal welfare and to minimize animal suffering in accordance with the recommendations of the Weatherall report on the use of nonhuman primates^52^. Animals were housed in adjoining individual primate cages, allowing social interactions, under controlled conditions of humidity, temperature, and light (12-h light/12-h dark cycles). Food and water were available ad libitum. Animals were monitored twice daily and fed commercial monkey chow, treats, and fruit twice daily by trained personnel.

### Human subjects

ICL was defined as having CD4 T cell counts of <300 μl/ml of blood at screening and on at least two occasions 6 weeks apart in the absence of any illness, treatment, or condition accounting for CD4 lymphopenia. All subjects with ICL as well as healthy control subjects were enrolled in clinical protocols approved by the National Institute of Allergy and Infectious Diseases institutional review board (NCT00867269, NCT00839436, and NCT00001281). All these protocols allow study procedures aimed to the collection and extensive immunological analysis of collected biological material. All subjects provided written informed consent prior to any study procedures and in accordance with the Declaration of Helsinki.

### Nonhuman primate sample collection

Blood, bronchoalveolar lavage, lymph nodes, and GI tissues were collected immediately post mortem. Mesenteric and axillary LN specimens for this study were maintained in RPMI and washed once in ice-cold phosphate-buffered saline. Loosely associated adipose and connective tissue were removed from the LN specimens, which were subsequently cut into roughly 1×1 mm^2^ sections. LN sections were digested through a 100 μm cell strainer and the resulting cell suspension was centrifuged for 5 min at 2500 rpm and washed twice in RPMI supplemented with 10% fetal bovine serum (FBS) (Hyclone, Logan, UT, USA) and penicillin/streptomycin antibiotic (cRPMI) (Life Technologies, Carlsbad, CA, USA). All LN cell suspensions were subsequently cryopreserved with freezing medium consisting of FBS and 10% dimethyl sulfoxide and stored in liquid nitrogen for later use.

### Flow cytometry

Polychromatic flow cytometry for immunophenotypic analysis was performed on stained LN cell suspensions using the BD LSRFortessa equipped with the FACS DiVA software (BD Biosciences, San Jose, CA, USA) and analyzed post acquisition using the FlowJo software (Treestar, Ashland, OR, USA). For each specimen, a minimum of three million cells were stained and a minimum threshold cutoff of 300 cells were acquired for the lowest-frequency ILC population. Specimens were stained with Live/dead AQUA dye (Invitrogen, Carlsbad, CA, USA) to assess cell viability and subsequently stained with company recommended concentrations with the following antibodies at 4 °C for 20 min: anti-CD45 Alexa Fluor 700 (clone F058-1283, BD Biosciences), anti-CD127 allophycocyanin-Cy7 (clone R34.34, Beckman Coulter, Brea, CA, USA), anti-CD117 Brilliant Violet 605 (clone 104D2, BioLegend, San Diego, CA, USA), anti-ST2 phycoerythrin (Polyclonal, R & D Systems, Minneapolis, MN, USA), anti-NKp44 phycoerythrin-vio770 (clone 2.29, Miltenyi, Auburn, CA, USA), anti-CD4 Brilliant Violet 711 (clone OKT4, BD Biosciences), anti-HLA-DR allophycocyanin (clone G46-6, BD Biosciences). The following antibodies, all conjugated to Brilliant Violet 421, were used in a lineage cocktail: anti-CD1a (clone HI149), anti-CD8 (clone RPA-T8), anti-CD11c (clone 3.9), anti-CD34 (clone 561), anti-CD123 (clone 6H6) (all BioLegend), anti-CD3 (clone SP34-2), anti-CD14 (clone M5E2), anti-CD16 (clone 3G8), anti-CD20 (clone L27), and anti-CD23 (clone M-L233) (All BD Biosciences). For intracellular antigens, after fixation and permeabilization (BD Biosciences Fix/Perm Kit), cells were stained with anti-ki67 fluorescein (clone B56, BD Biosciences), anti-active Caspase-3 Brilliant Violet 650 (clone C92-605, BD Biosciences), and anti-granzyme B phycoerythrin Texas Red (clone GB11, Thermo Fisher, Waltham, MA, USA). Transcription factor expression was assessed with Foxp3/Transcription factor buffers (eBioscience, San Diego, CA, USA), staining cell suspensions with anti-GATA-3 phycoerythrin CF594 (clone L50-823, BD Biosciences), and anti-RORγt allophycocyanin (clone AFKJS-9, eBioscience). To assess distinct cytokine profiles of ILCs, cell suspensions were stimulated for 6 h at 37 °C with 50 ng/ml PMA and 1 μg/ml ionomycin (both from Sigma-Aldrich, St. Louis, MO, USA) in the presence of 10 μg/ml Brefeldin A (BD Biosciences). Cell suspensions were fixed, permeabilized, and stained with anti-TNF Brilliant Violet 786 (clone Mab11, BioLegend), anti-IL-13 fluorescein (clone 85BRD, eBioscience), anti-IL-17A Brilliant Violet 711 (clone BL168, BioLegend), and anti-IL-22 allophycocyanin (clone IL22JOP, eBioscience). Identical antibodies were used to assess ILCs in human PBMC, with the addition of anti-CRTH2 phycoerythrin cy7 (clone BM16, BioLegend), and anti-CD161 Brilliant Violet 786 (clone HP-3G10, BioLegend).

### Cell sorting and in vitro culture of ILCs

Four-way sorts were performed on stained cell suspensions using a BD FACSAria equipped with FACS DiVa software (BD Biosciences). ILC populations were sorted according to the gating strategy depicted in Fig. 1a. Cell populations were sorted into collection tubes containing 500 μl of FBS and subsequently spun down at 2500 rpm for 8 min. Sorted cell populations were suspended and cultured at a minimum cell density of 1000 cell/200 μl cRPMI in a 96-well plate in the presence of 25 ng/ml recombinant human interleukin-7 (IL-7) (Peprotech, Rocky Hill, NJ, USA) for 6 days at 37 °C. In separate conditions, 10 ng/ml recombinant human IL-1β (R & D systems) and/or 500 U/ml recombinant rhesus IFN-α2 (PBL Assay Science, Piscataway, NJ, USA) were added.

### Whole-transciptome library preparation, sequencing, and processing

ILC supopulations from cryopreserved MLN cell suspensions were sorted directly into RLT lysis buffer (Qiagen, Hilden, Germany). RNA was isolated using RNeasy Micro spin columns (Qiagen, Germantown, MD, USA) and measured for quality using a Fragment Analyzer (Advanced Analytical Technologies Inc., Ankeny, IA, USA). Complementary DNA were prepared using the SMART-Seq v4 Ultra Low Kit (Clontech, Mountain View, CA, USA) and sequencing libraries were prepared with the Nextera XT DNA Preparation Kit (Illumina, San Diego, CA, USA), using the manufacturer’s standard protocols. Library quality was assessed by measuring size and distribution using a Fragment Analyzer (average size: 509.4 bp; size distribution: ~150–2500 bp) and library concentration determined by Qubit 3.0 fluorometric analysis (Thermo Fisher Scientific, Waltham, MA, USA). The libraries were run on a HiSeq 2500 with 125 bp paired end reads (average reads per sample: 36.61 million). Reads were trimmed of Illumina adapter sequences using Skewer v0.2.2. Trimmed paired end reads were mapped against the macaque genome (MMUL 8.0.1, v102) using the HISAT2 aligner v2.0.3 and sorted with SAMtools v1.3.1. Transcript abundances were estimated using featureCounts v1.5.0-p2. The count matrices were then normalized and tested for DEGs using edgeR v3.12.1 and its generalized linear model likelihood ratio test. Pathway enrichment between groups was performed on the normalized count matrices using GSVA v1.18.0 and a moderated Student’s *t* test from limma v3.26.9. Sequence and gene expression data are available at the Gene Expression Omnibus, accession number GSE116013.

### Fluorescence microscopy and quantitative image analysis

Formaldehyde-fixed, paraffin-embedded mesenteric lymph node tissues sections (5 μm thick) were placed on Fisher-Plus microscope slides, deparaffinized in xylenes, rehydrated through graded ethanols, and subjected to heat-induced epitope retrieval (HIER) buffer with 0.1% citraconic anhydride (Sigma-Aldrich; 125318), cooled, washed in double-distilled H_2_O (ddH_2_O) and treated with proteinase K treatment (2 μg/ml) for 10 min (Fisher Scientific; BP1700). The slides were washed in ddH_2_O, blocked with 0.25% casein in Tris buffer, and sequentially stained. For CD117 detection, staining was performed with a 1:80 dilution overnight at 4 °C (Sigma-Aldrich; HPA004471), and detection was performed with an anti-rabbit polymer horseradish peroxidase (HRP)-conjugated system (GBI Labs; D39-110) and developed with Alexa Fluor 488-conjugated tyramide at a 1:1000 dilution for 10 min at room temperature (Invitrogen; B40953). Antibody stripping was performed following detection of CD117 by microwaving slides in 0.1% citraconic anhydride HIER buffer for 60 s at full power followed immediately for 15 min at 20% power. Slides were cooled to room temperature for 20 min, washed in ddH_2_O, blocked with 0.25% casein in Tris buffer, and stained for CD3 (Dako; clone F7.2.38), using a 1:100 dilution for 1 h at room temperature, followed by an anti-mouse polymer HRP-conjugated system (GBI Labs; D12-110), in conjunction with Alexa Fluor 594-conjugated tyramide at a 1:1000 dilution for 10 min at room temperature (Invitrogen; B40957). After washing in Tris-buffered saline, the slides were stained with DAPI (4′,6-diamidino-2-phenylindole; Roche; 10 236 276 001), used at 1:1000 dilution, for 15 min to visualize nuclei. Imaging of the paracortical T cell zone was performed on an Olympus Fluoview FV10i confocal laser scanning microscope using a ×60 phase contrast oil-immersion objective (NA 1.35) imaging using sequential mode to separately capture the fluorescence from the different fluorochromes at an image resolution of 1024 × 1024 pixels. Quantification was performed by selecting 10 random fields (210 μm × 210 μm) per slide within the cortex/paracortex and manually quantifying the CD117^+^ CD3^−^ cells within each field.

### Statistics

Statistical analyses were performed using Prism (v6.0; GraphPad Software, La Jolla, CA, USA). The Mann–Whitney *U* test was used for comparisons between groups. A paired Student’s *t* test was used to compare treatment differences in vitro within same donors. Correlations were determined using Spearman's rank coefficients and significance was determined using linear regression analysis. *P* values <0.05 were considered significant. Graphical representation of significance is as follows: ns = *P* > 0.05, * = *P* ≤ 0.05, ** = *P* ≤ 0.01, *** = *P* ≤ 0.001, and **** = *P* ≤ 0.0001. Summary data are presented as dot plots with median lines.

### Data availability

All relevant data, including .fcs files, .fastq files, scripts used for alignment (HISAT2), differential gene expression (EdgeR), and tertiary data analysis are available from the authors upon request. Sequence and gene expression data are available at the NCBI Sequence Resource Archive and Gene Expression Omnibus, respectively. Accession numbers are pending.

## Electronic supplementary material

Supplementary Information

## References

[1] Brenchley JM (2006). Microbial translocation is a cause of systemic immune activation in chronic HIV infection. Nat. Med..

[2] Estes JD (2010). Damaged intestinal epithelial integrity linked to microbial translocation in pathogenic simian immunodeficiency virus infections. PLoS. Pathog..

[3] Mattapallil JJ (2005). Massive infection and loss of memory CD4+ T cells in multiple tissues during acute SIV infection. Nature.

[4] Pandrea IV (2007). Acute loss of intestinal CD4+ T cells is not predictive of simian immunodeficiency virus virulence. J. Immunol..

[5] Hao XP (2015). Experimental colitis in SIV-uninfected rhesus macaques recapitulates important features of pathogenic SIV infection. Nat. Commun..

[6] Freeman ML (2016). Reply to Barrett, et al. Clin. Infect. Dis..

[7] Lederman MM, Funderburg NT, Sekaly RP, Klatt NR, Hunt PW (2013). Residual immune dysregulation syndrome in treated HIV infection. Adv. Immunol..

[8] Cecchinato V (2008). Altered balance between Th17 and Th1 cells at mucosal sites predicts AIDS progression in simian immunodeficiency virus-infected macaques. Mucosal Immunol..

[9] Macal M (2008). Effective CD4+ T-cell restoration in gut-associated lymphoid tissue of HIV-infected patients is associated with enhanced Th17 cells and polyfunctional HIV-specific T-cell responses. Mucosal Immunol..

[10] Raffatellu M (2008). Simian immunodeficiency virus-induced mucosal interleukin-17 deficiency promotes Salmonella dissemination from the gut. Nat. Med..

[11] Ryan ES (2016). Loss of function of intestinal IL-17 and IL-22 producing cells contributes to inflammation and viral persistence in SIV-INFECTED RHESUS MACAQues. PLoS. Pathog..

[12] Schuetz A (2014). Rv254/Search, R. S. S. Groups, Initiation of ART during early acute HIV infection preserves mucosal Th17 function and reverses HIV-related immune activation. PLoS Pathog..

[13] Hazenberg MD, Spits H (2014). Human innate lymphoid cells. Blood.

[14] Cella M (2009). A human natural killer cell subset provides an innate source of IL-22 for mucosal immunity. Nature.

[15] Zhang Z (2015). Plasmacytoid dendritic cells promote HIV-1-induced group 3 innate lymphoid cell depletion. J. Clin. Invest..

[16] Hepworth MR (2013). Innate lymphoid cells regulate CD4+ T-cell responses to intestinal commensal bacteria. Nature.

[17] Sonnenberg GF (2012). Innate lymphoid cells promote anatomical containment of lymphoid-resident commensal bacteria. Science.

[18] Kloverpris HN (2016). Innate lymphoid cells are depleted irreversibly during acute HIV-1 infection in the absence of viral suppression. Immunity.

[19] Xu H (2012). IL-17-producing innate lymphoid cells are restricted to mucosal tissues and are depleted in SIV-infected macaques. Mucosal Immunol..

[20] Reeves RK (2011). Gut inflammation and indoleamine deoxygenase inhibit IL-17 production and promote cytotoxic potential in NKp44+ mucosal NK cells during SIV infection. Blood.

[21] Li H (2014). Hypercytotoxicity and rapid loss of NKp44+ innate lymphoid cells during acute SIV infection. PLoS Pathog.

[22] Xu H, Wang X, Lackner AA, Veazey RS (2015). Type 3 innate lymphoid cell depletion is mediated by TLRs in lymphoid tissues of simian immunodeficiency virus-infected macaques. FASEB J..

[23] Forkel M, Mjosberg J (2016). Dysregulation of group 3 innate lymphoid cells in the pathogenesis of inflammatory bowel disease. Curr. Allergy Asthma Rep..

[24] Cupedo T (2009). Human fetal lymphoid tissue-inducer cells are interleukin 17-producing precursors to RORC+ CD127+ natural killer-like cells. Nat. Immunol..

[25] Hoyler T (2012). The transcription factor GATA-3 controls cell fate and maintenance of type 2 innate lymphoid cells. Immunity.

[26] Vely F (2016). Evidence of innate lymphoid cell redundancy in humans. Nat. Immunol..

[27] Simoni Y (2017). Human innate lymphoid cell subsets possess tissue-type based heterogeneity in phenotype and frequency. Immunity.

[28] Bjorklund AK (2016). The heterogeneity of human CD127(+) innate lymphoid cells revealed by single-cell RNA sequencing. Nat. Immunol..

[29] Silver JS (2016). Inflammatory triggers associated with exacerbations of COPD orchestrate plasticity of group 2 innate lymphoid cells in the lungs. Nat. Immunol..

[30] Lombardi V (2016). Circulating innate lymphoid cells are differentially regulated in allergic and nonallergic subjects. J. Allergy Clin. Immunol..

[31] Smith SG (2016). Increased numbers of activated group 2 innate lymphoid cells in the airways of patients with severe asthma and persistent airway eosinophilia. J. Allergy Clin. Immunol..

[32] Klatt NR (2012). Loss of mucosal CD103+ DCs and IL-17+ and IL-22+ lymphocytes is associated with mucosal damage in SIV infection. Mucosal Immunol..

[33] Bal SM (2016). IL-1beta, IL-4 and IL-12 control the fate of group 2 innate lymphoid cells in human airway inflammation in the lungs. Nat. Immunol..

[34] Bernink JH (2013). Human type 1 innate lymphoid cells accumulate in inflamed mucosal tissues. Nat. Immunol..

[35] Bernink JH (2015). Interleukin-12 and -23 control plasticity of CD127(+) group 1 and group 3 innate lymphoid cells in the intestinal lamina propria. Immunity.

[36] Zonios D, Sheikh V, Sereti I (2012). Idiopathic CD4 lymphocytopenia: a case of missing, wandering or ineffective T cells. Arthritis Res. Ther..

[37] Sandler NG (2011). Plasma levels of soluble CD14 independently predict mortality in HIV infection. J. Infect. Dis..

[38] Satoh-Takayama N (2010). IL-7 and IL-15 independently program the differentiation of intestinal CD3-NKp46+ cell subsets from Id2-dependent precursors. J. Exp. Med..

[39] Kramer B (2017). Compartment-specific distribution of human intestinal innate lymphoid cells is altered in HIV patients under effective therapy. PLoS. Pathog..

[40] Colle JH (2006). Regulatory dysfunction of the interleukin-7 receptor in CD4 and CD8 lymphocytes from HIV-infected patients—effects of antiretroviral therapy. J. Acquir. Immune Defic. Syndr..

[41] Rethi B (2005). Loss of IL-7Ralpha is associated with CD4 T-cell depletion, high interleukin-7 levels and CD28 down-regulation in HIV infected patients. AIDS.

[42] Monticelli LA (2011). Innate lymphoid cells promote lung-tissue homeostasis after infection with influenza virus. Nat. Immunol..

[43] Bouchery T (2015). ILC2s and T cells cooperate to ensure maintenance of M2 macrophages for lung immunity against hookworms. Nat. Commun..

[44] Geremia A (2011). IL-23-responsive innate lymphoid cells are increased in inflammatory bowel disease. J. Exp. Med..

[45] Sawa S (2011). RORgammat+ innate lymphoid cells regulate intestinal homeostasis by integrating negative signals from the symbiotic microbiota. Nat. Immunol..

[46] Vaccari M (2016). Adjuvant-dependent innate and adaptive immune signatures of risk of SIVmac251 acquisition. Nat. Med..

[47] Barouch DH (2016). Rapid inflammasome activation following mucosal SIV infection of rhesus monkeys. Cell.

[48] Liyanage NP (2014). Antiretroviral therapy partly reverses the systemic and mucosal distribution of NK cell subsets that is altered by SIVmac(2)(5)(1) infection of macaques. Virology.

[49] Weizman OE (2017). ILC1 confer early host protection at initial sites of viral infection. Cell.

[50] Wu F (2012). Sequential evolution and escape from neutralization of simian immunodeficiency virus SIVsmE660 clones in rhesus macaques. J. Virol..

[51] Goldstein S (1994). Immunization with whole inactivated vaccine protects from infection by SIV grown in human but not macaque cells. J. Med. Primatol..

[52] Arnason G (2017). The ethical justification for the use of nonhuman primates in research: the Weatherall report revisited. J. Med. Ethics.

