# Supplementary material for: Hallmarks of primate lentiviral immunodeficiency infection recapitulate loss of innate lymphoid cells
Source: Nat Commun. 2018 Sep 27;9:3967. doi: 10.1038/s41467-018-05528-3 (PMC6160474; doi:10.1038/s41467-018-05528-3)
Supplement: Supplementary file 1 — Supplementary Information [file 41467_2018_5528_MOESM1_ESM.pdf]

Supplementary information

Hallmarks of primate lentiviral immunodeficiency infection recapitulate loss of innate lymphoid cells

Mudd, et al.

Supplementary figure 1

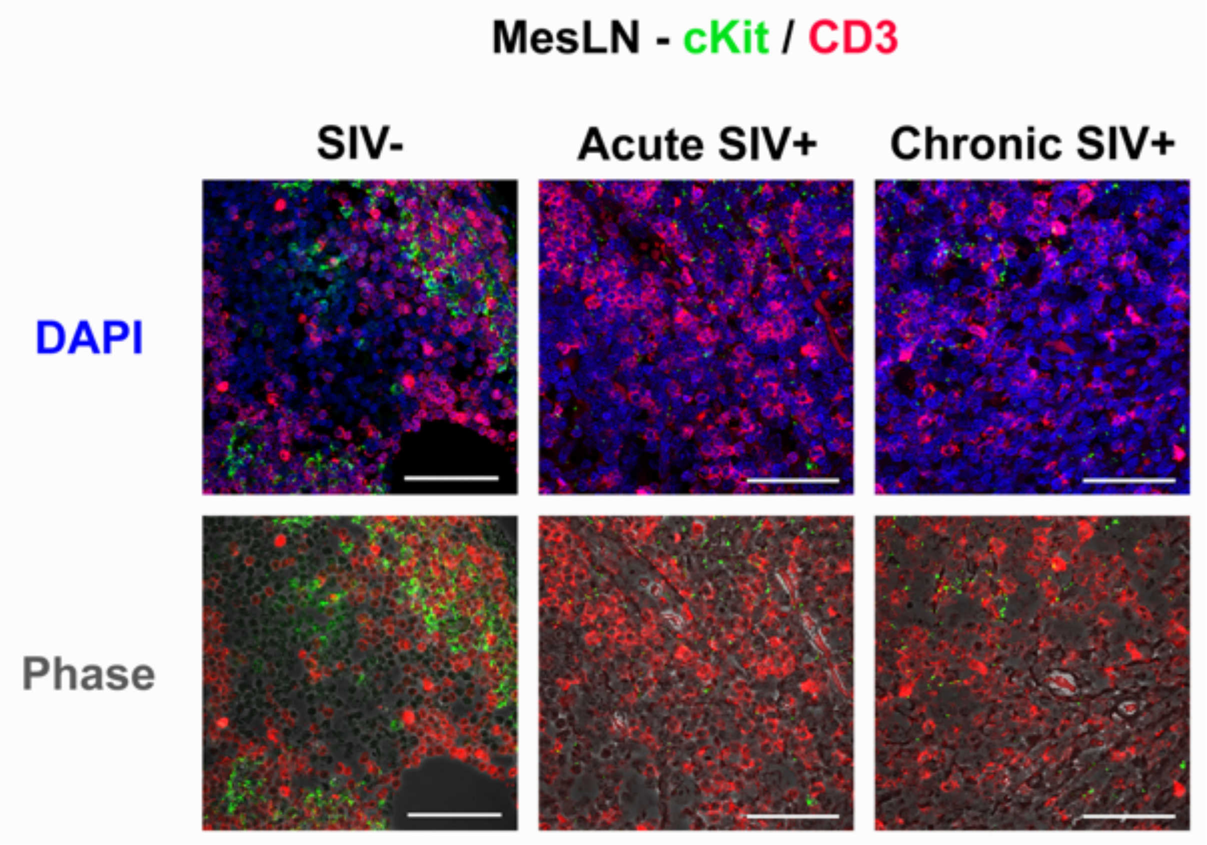

Supplementary figure 1 CD3-cKit+ cells are depleted numerically in the SIV+ MLN. Formaldehyde fixed, paraffin-embedded mesenteric lymph node tissue sections were stained for cKit, CD3 and DAPI to visualize cell nuclei. Non-stromal CD3-cKit+ staining was apparent in the LN paracortex and quantification of these cells was performed by selecting 10 random fields (210µm X 210µm) per slide. Micoscopy pictures are representative of healthy uninfected, acute and chronic SIV+ MLNs. Scale bars= 50µm.

Supplementary figure 2

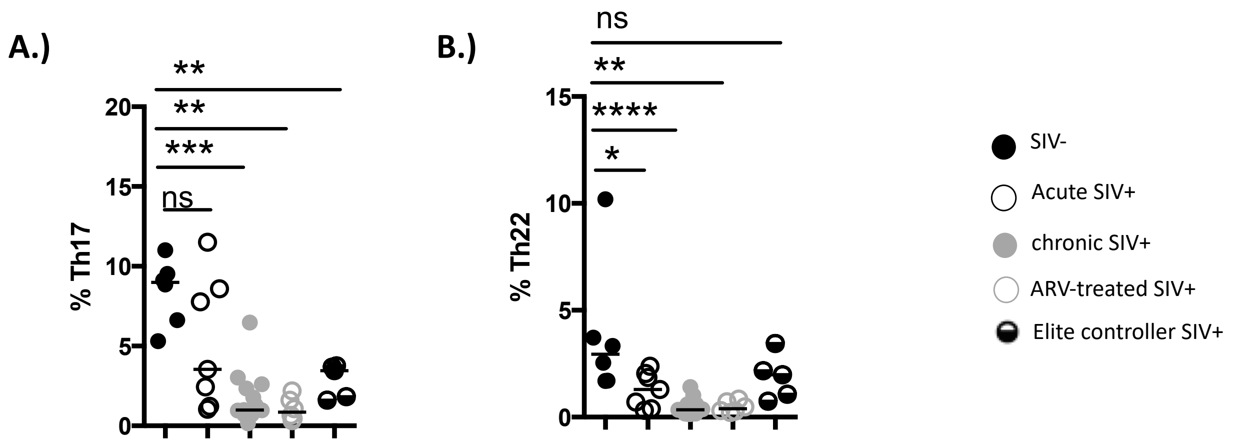

Supplementary figure 2. Th17 and Th22 cells are diminished in the SIV+ MLN. MLN cell suspensions were stimulated with PMA/Ionomycin under the presence of Brefeldin A and intracellular IL-17 (A) and IL-22 (B) were assessed after 6 hours in SIV- , acute SIV+, chronic SIV+, SIV+ RMs administered ART in chronic SIV infection, and elite controller RMs. Statistical significance was determined by the Mann-Whitney test.

Supplemental Figure 3

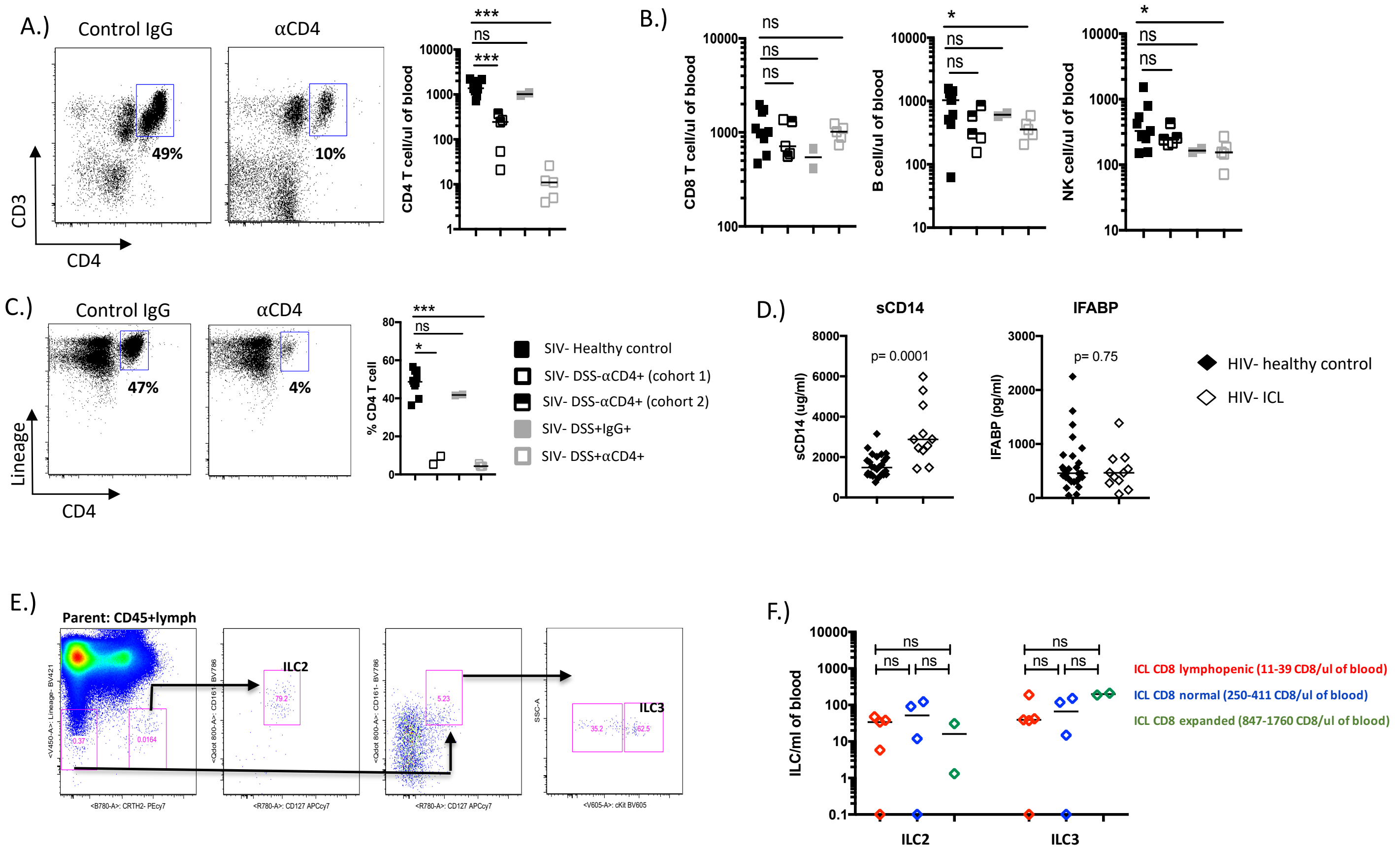

Supplemental figure 3. αCD4 treatment diminishes CD4+ T cells in tissues with minimal effect on other leukocyte subsets. (A) CD4+ T cell numbers in blood with representative histograms of two cohorts of RMs receiving αCD4 , DSS, or both treatments. **(B)** Numbers of CD8+ T cell, B cell, and NK cells in blood of RMs receiving αCD4, DSS, or both treatments. (**C**) representative histograms and summary data of MLN CD4+ T cell proportions in healthy control, αCD4, DSS, or animals receiving both treatments. (**D**) measurments of soluble proteins sCD14 and IFABP in plasma. (**E**). Representative gating strategy to define human ILCs in peripheral blood. (**F**) Within the cohort of healthy control subjects (N=11), the range of CD8 T cell count was determined. ICL subjects falling below the range of healthy control CD8 counts were defined as "ICL CD8 lymphopenic", within the CD8 count range of healthy subjects as "ICL CD8 normal", and above the CD8 count range as "ICL CD8 lymphocytosis". A mann-whitney test was used to determine significance.

Supplementary figure 4

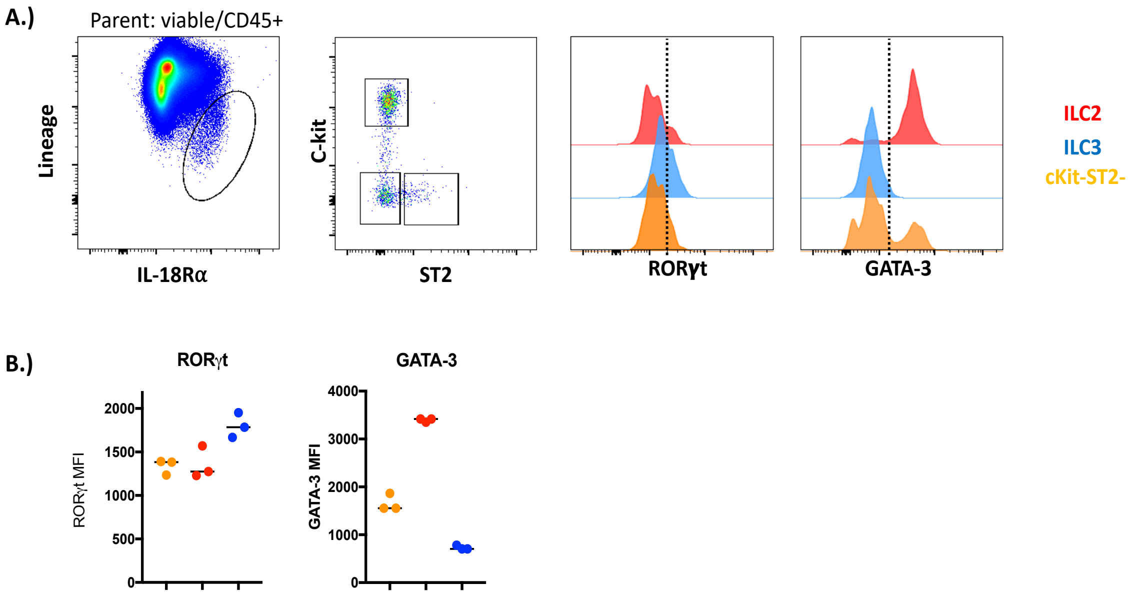

Supplementary figure 4. **Transcription factor staining in IL-18Rα+ ILCs**. (**A**) Representative gates defining IL-18R+ and lineage-defining transcription factors in the MLN of RMs. (**B**) Summary data of GATA-3 and RORgT transcription factor MFI in ILC2, ILC3, and cKit-ST- populations.

Supplementary figure 5

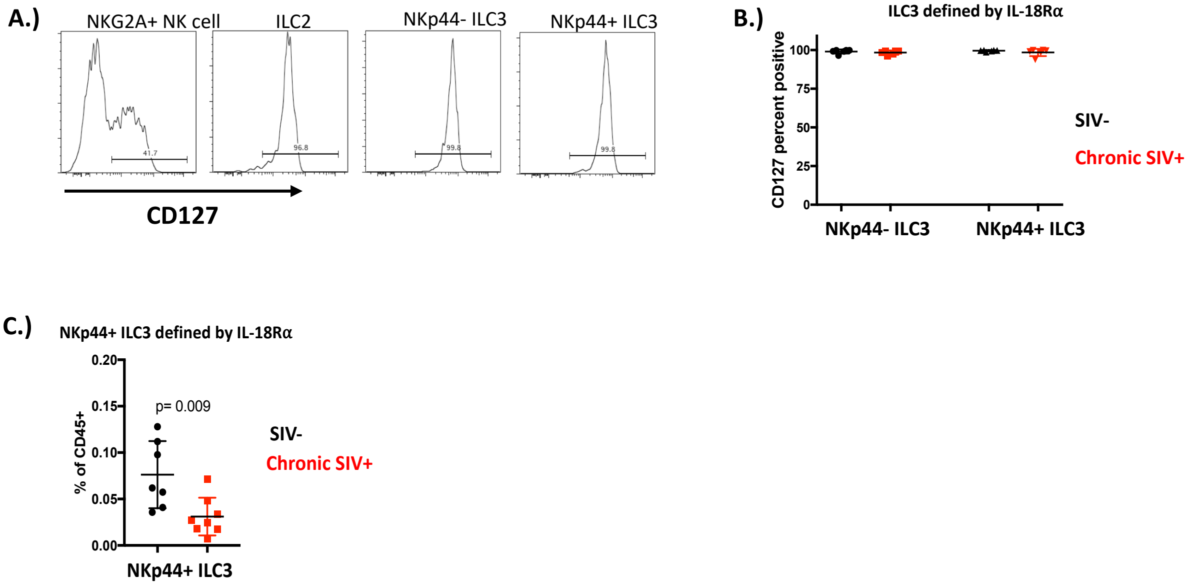

Supplementary figure 5. Despite lower surface densities, CD127 expression is not lost in ILC3s of the SIV+ MLN. (A) representative histogram of CD127 surface expression onIL-18R+ ILC subtypes, relative to CD127-negative NKG2A NK cells. **(B)** CD127 expression representative as percent positive in IL-18R+ ILC3s. Gate is set relative to CD127-negative NKG2A NK cells. (**C**) Summary data of NKp44+ ILC3 proportions defined by IL-18R in the healthy uninfected and chronically SIV-infected MLN. Significiance in (C) was determined by Mann-Whitney test.

Supplementary Table 1. Demographic, Clinical and Peripheral Blood Immunophenotyping of human subjects

Group	Subject ID	Age	Lymphocytes		CD4		CD8		NK		Infectious Complications
			%	Cells/μL	%	Cells/μL	%	Cells/μL	%	Cells/μL	
ICL	ICL1	58	9.9	570	3.7	22	4.3	26	19.3	106	Disseminated histoplasmosis
	ICL2	44	17.1	750	32.3	242	37.4	280	11.6	87	Upper respiratory tract infections
	ICL3	55	12.1	600	1.8	11	5.9	35	21.6	130	None
	ILC4	59	44.1	2100	2.8	59	83.8	1760	4.7	99	Cryptococcal meningitis, severe HPV-related dysplasia
	ICL5	56	16.1	740	5.5	41	55.6	411	19.1	141	Cryptococcal meningitis
	ICL6	35	8.6	450	9	2.1	2.9	13	30.9	139	Recalcitrant HPV-related warts
	ICL7	58	24.4	1230	7.7	95	68.9	847	8	98	Cryptococcal meningitis
	ICL8	55	11.5	470	2.7	13	2.4	11	19.3	91	Cutaneous mycobacterium avium, molluscum contagiousum
	ICL9	66	19.2	780	34.4	268	37.3	291	9	70	None
	ICL10	51	9.7	380	0.5	2	15.3	58	35.8	50	Cryptococcal meningitis
	ICL11	67	20.7	920	26.8	247	17.2	158	30.2	278	None
HC	HC1	47	27	1976	44	870	36	712	4	79	NA
	HC2	46	24	1810	43	778	33	597	6	109	NA
	HC3	41	18	1209	53	641	25	302	14	169	NA
	HC4	66	29	1849	54	998	28	518	10	185	NA
	HC5	39	21.7	1708	46	786	16	273	16	273	NA
	HC6	40	27.2	1750	47.4	830	20	350	17.8	312	NA
	HC7	39	28	1279	47	601	18	230	20	914	NA
	HC8	57	29	1325	57	716	15	188	12	520	NA
	HC9	48	37	1332	38	505	25	332	13	468	NA

Supplementary Table 2. ILCs in peripheral blood of human subjects

Group	Subject ID	ILC1		ILC2		ILC3	
		**%**	**cells/mL**	**%**	**cells/mL**	**%**	**cells/mL**
ICL	ICL1	0.00E+00	0.00E+00	0.00E+00	0	0.00E+00	0
	ICL2	2.89E-03	2.17E+01	0.0122	91.5	0.0179	134.25
	ICL3	4.60E-03	2.76E+01	5.99E-04	3.594	0.026	156
	ILC4	1.33E-03	2.79E+01	1.62E-03	34.02	0.0111	233.1
	ICL5	6.57E-04	4.86E+00	1.48E-03	10.952	0.0174	128.76
	ICL6	1.49E-02	6.71E+01	8.54E-03	38.43	8.54E-03	38.43
	ICL7	0	0.00E+00	2.54E-04	3.1242	1.52E-03	18.696
	ICL8	2.40E-03	1.13E+01	7.56E-03	35.532	6.87E-03	32.289
	ICL9	2.11E-03	1.65E+01	0.0136	106.08	3.83E-03	29.874
	ICL10	0	0.00E+00	0	0	0	0
	ICL11	9.12E-04	8.39E+00	0.0527	484.84	8.06E-03	74.152
HC	HC1	1.55E-03	3.06E+01	0.025	494	0.0212	418.912
	HC2	1.62E-03	2.93E+01	0.0468	847.08	0.0235	425.35
	HC3	2.42E-03	2.93E+01	0.201	2430.09	0.0418	505.362
	HC4	2.10E-03	3.88E+01	0.0115	212.635	0.0111	205.239
	HC5	2.60E-03	4.44E+01	0.0239	408.212	0.0287	490.196
	HC6	1.53E-03	2.68E+01	0.0256	448	1.75E-02	306.25
	HC7	2.87E-03	3.67E+01	0.011	140.69	8.42E-03	107.6918
	HC8	9.21E-04	1.22E+01	0.0206	272.95	8.14E-03	107.855
	HC9	6.04E-03	8.05E+01	0.044	586.08	0.0263	350.316
